# Respiratory Syncytial Virus Epidemiology and Clinical Characteristics among Young Children Hospitalized in Sierra Leone

**DOI:** 10.4269/ajtmh.24-0845

**Published:** 2025-09-04

**Authors:** Foday U. Turay, Robert J. Samuels, Gustavo Amorim, Donald S. Grant, Natasha B. Halasa, John S. Schieffelin, Troy D. Moon

**Affiliations:** ^1^ College of Medicine and Allied Health Sciences, University of Sierra Leone, Free Town, Sierra Leone;; ^2^ Department of Tropical Medicine and Infectious Diseases, Tulane University Celia, Scott Weatherhead School of Public Health and Tropical Medicine, New Orleans, Louisiana;; ^3^ Kenema Government Hospital, Ministry of Health and Sanitation, Kenema, Sierra Leone;; ^4^ Department of Biostatistics, Vanderbilt University Medical Center, Nashville, Tennessee;; ^5^ Department of Pediatrics, Division of Pediatric Infectious Diseases, Vanderbilt University Medical Center, Nashville, Tennessee;; ^6^ Department of Pediatrics, Division of Pediatric Infectious Diseases, Tulane University, New Orleans, Louisiana

## Abstract

Respiratory syncytial virus (RSV) is a leading cause of acute lower respiratory tract infections in children under 5 years of age, resulting in significant morbidity and mortality worldwide. The aim of the current study is to investigate the prevalence and clinical features of RSV disease in hospitalized infants in Sierra Leone. A prospective study was conducted on children under 2 years of age who were hospitalized at Kenema Government Hospital between October 1, 2020, and January 31, 2023. A total of 912 children participated in the study, with 147 (22.8%) testing positive for RSV of 644 (70.6%) who tested positive for at least one virus. During the rainy seasons of 2021 and 2022 (May to November), a surge in RSV cases was observed, particularly those attributed to RSV-A. Conversely, during the dry season (December to April), RSV activity was relatively lower. Respiratory syncytial virus B was significantly associated with a higher disease severity score and increased likelihood of requiring oxygen therapy or referral to the intensive care unit (ICU). Younger children infected with RSV were significantly more likely to require oxygen therapy or referral to the ICU and exhibit higher severity scores. In conclusion, the current study provides valuable insights into the epidemiology and clinical characteristics of RSV in hospitalized children under 2 years of age in Sierra Leone. These findings highlight the need for continuous surveillance and monitoring of RSV infections, especially during peak and transitional seasons, to inform public health interventions and reduce the burden of RSV on children’s health.

## INTRODUCTION

Respiratory syncytial virus (RSV) is a significant global health problem because it greatly contributes to the occurrence of acute lower respiratory tract infections in children under 5 years of age.^1^^,^^2^ Respiratory syncytial virus infections exhibit a wide range of manifestations, from mild, cold-like symptoms to severe respiratory distress.^3^ Clinical presentations often lead to recurrent wheezing, bronchiolitis, pneumonia, and hospitalization, particularly in children under 2 years of age.^4^ Severe RSV-related illness is more likely to affect infants born prematurely and those with underlying health conditions, such as chronic lung disease, congenital heart defects, or immunodeficiency disorders;^5^^,^^6^ however, the majority of infants hospitalized with RSV do not have an underlying medical condition. Severe RSV in early life can potentially increase the likelihood of developing childhood asthma in certain infants.^7^

Sierra Leone, located in West Africa, has an estimated population of 8.6 million people as of 2022 and was ranked 184th of 193 countries on the 2023/2024 United Nations Development Program Human Development Index.^8^ Sierra Leone’s healthcare system has been severely undermined due to decades of national development instability, which includes a longstanding civil war that occurred between 1991 and 2002 and the West African Ebola outbreak that took place from 2013 to 2015.^9^ Since 2006, Sierra Leone has participated in national-level influenza surveillance and has begun contributing to the WHO Strengthening Influenza Sentinel Surveillance in Africa network in 2011.^10^^,^^11^ Other than these data, there remains a paucity of information in Sierra Leone related to the prevalence, temporality, and epidemiology of the most common and emerging human respiratory viruses, including RSV.^12^

Respiratory syncytial virus is categorized into two primary subtypes (RSV-A and RSV-B) according to variations in their genetic and antigenic characteristics. These subtypes commonly circulate together and result in outbreaks; however, they frequently vary in their prevalence and the severity of disease presentation.^13^ Understanding these distinctions at a national and hyperlocal level (i.e., region or community) is crucial for establishing efficient measures to prevent and treat illness.

The objective of the present study is to describe the epidemiology, seasonality, and clinical characteristics of RSV in children under 2 years of age at Kenema Government Hospital (KGH) in Kenema, Sierra Leone, between October 2020 and January 2023. An additional aim of this study is to provide a more detailed description of the relative contribution of each individual RSV subtype. By documenting the prevalence and clinical characteristics of RSV infection over a 28-month period spanning three consecutive RSV transmission seasons, the aim is to provide insights that can inform the development of public health strategies and clinical management practices for RSV infections in children in Sierra Leone.

## MATERIALS AND METHODS

### Design and population of the study.

A prospective respiratory virus surveillance study was conducted among young children who were hospitalized at KGH between October 1, 2020, and January 31, 2023. The study methods have been previously described, including a comprehensive list of all viruses tested.^12^ Children were eligible for enrollment if they were less than 24 months of age, had been admitted to the hospital within 48 hours before enrollment, and had a history of respiratory symptoms, such as nasal flaring, cough (<14 days), elevated respiratory rate based on age, or difficulty breathing. Children under 1 month of age were excluded from participation. A child could only be enrolled in the present study once; they were also excluded if they were hospitalized for subsequent respiratory illnesses during the study period.

### Study setting.

**Figure 1.**
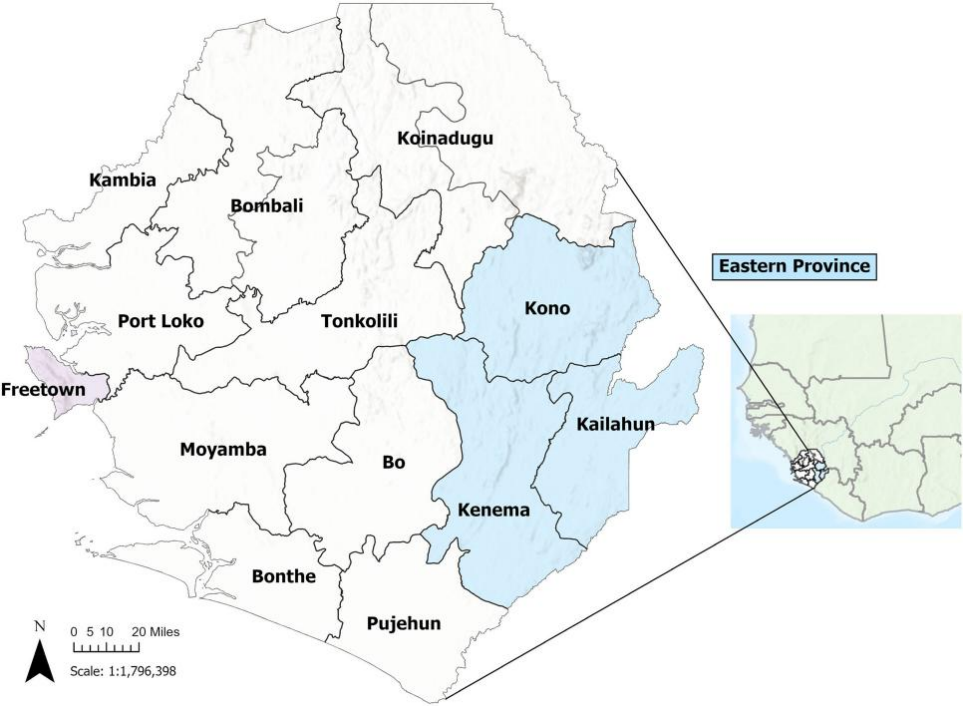
Map of Sierra Leone, with Eastern Province highlighted. *Designed by Katherine A. Gruber.

The present study took place at KGH, a hospital in Sierra Leone’s Kenema District in Eastern Province, whose pediatric catchment area suffers from a high burden of infectious diseases (Figure 1).^12^^,^^14^^,^^15^ The primary factors leading to hospitalization among children under 5 years old at KGH include malaria, pneumonia, malnutrition, and gastrointestinal diseases. The pediatric ward of KGH provides free medical care to children aged 5 years or younger, with an average monthly admission of ∼400 children. Kenema Government Hospital predominantly utilizes the Emergency Triage, Assessment, and Treatment Plus protocol for triaging pediatric patients who present to the hospital’s emergency department or urgent care.^16^

### Enrollment and data collection.

The emergency care and triage nurses performed pre-eligibility assessments for all children with acute respiratory symptoms. Once the initial eligibility was established, study personnel approached parents or legal guardians to discuss enrollment until a daily limit of three new patients per day was reached. Enrollment took place from 6 am to 6 pm, Monday through Friday. This daily quota enrollment strategy ensured that a controlled and consistent number of patients were enrolled across multiple study months, allowing for the analysis of virus seasonality over time.

The study team collected data from patients using a paper-based instrument and a standardized case report form (CRF). The data were subsequently entered into an online Research Electronic Data Capture (REDCap, Nashville, TN) database. The CRF covered seven domains: introduction and screening, demographics and social history, current illness, diet and nutrition, physical examination, and laboratory results. At enrollment, participants’ respiratory symptom severity was evaluated using an adapted version of the bronchiolitis severity score published by Tal et al. The original severity score variables included respiratory rate; wheezing; flaring, retractions, and accessory muscle use; and cyanosis. In the current study’s adaptation, cyanosis was removed, and oxygen saturation on room air was added. In addition, wheezing and nasal flaring, as well as the use of accessory muscle variables, were collapsed into a dichotomous “yes” is present/“no” is not present response. Patients were subsequently classified as having mild, moderate, or severe symptoms (Supplemental File 1).^17^ After admission, discharge, referral, or death, patient records were thoroughly reviewed, including demographic details, medication, and medical history, as well as comprehensive clinical course information. The study team reviewed the completed paper-based study instruments and verified the data entered into the electronic database as part of the data quality control process.

### Laboratory procedures.

Nasopharyngeal and oropharyngeal swabs were collected for each participant at enrollment using a Puritan Hydraflock nose swab 25–3320-H EMB 80MM and a Puritan Hydraflock oral swab (Puritan™ 253406H; Thermo Fisher Scientific, Waltham, MA). Samples were stored at –80°C at KGH until they could be transported on dry ice to Vanderbilt University Medical Center for analysis. Total nucleic acid was purified from aliquoted specimens as previously described, and extracts were tested for 11 respiratory pathogen targets using the NxTAG Respiratory Pathogen Panel + Severe Acute Respiratory Syndrome Coronavirus 2 (SARS-CoV-2; Luminex, Austin, TX).^12^

## STATISTICAL ANALYSES

The present analysis was focused on children identified as being RSV positive upon admission to the hospital and further subtyped as having either RSV-A or RSV-B. Descriptive statistics are presented as frequencies (percentages) or medians and interquartile ranges (IQRs), when appropriate. Categorical variables were compared using Pearson χ^2^ tests. Continuous variables were compared using the Mann–Whitney *U* test. Multivariable logistic regression was used to identify risk factors associated with a higher likelihood of requiring oxygen (O^2^) therapy, being referred to the intensive care unit (ICU), and an increasing disease severity score. Variables were chosen a priori on the basis of the existing literature and clinical expertise. The severity score was treated as an ordinal, continuous variable, and a multivariable ordinal regression model with a logit link was used to assess its relationship with covariates. A binary variable was established to identify patients with specific viruses. The statistical analysis was conducted using R version 4.2.0 (R Foundation, Vienna, Austria).^18^

## RESULTS

### Demographic and clinical characteristics.

From October 1, 2020 to January 31, 2023, we enrolled 912 children under 24 months of age. Of these, 644 (70.6%) tested positive for at least one virus, and of those, 147 (22.8%) were confirmed to be positive for RSV. Among the 644 children with at least one virus detected, only 29 (4.5%) were diagnosed with SARS-CoV-2 (data not shown). Of the RSV-positive children, 45 (36%) also had another virus detected, with no co-detection of SARS-CoV-2.

Of RSV-positive children, 54% were female, and the median age at enrollment was 5.0 months (IQR: 2.7 to 10.4; Table 1). More than 80% of RSV-positive children were born prematurely (<37 weeks’ gestational age), and the reported length of exclusive breastfeeding was a median of 5.0 months (IQR: 2.0 to 9.0). Indoor pollutant exposure was observed among RSV-positive children in the present study, with 27.2% of the cohort reporting a smoker living in the household, and 7.5% indicating that their household used an indoor burning stove. When demographic characteristics were compared across the different RSV subtypes, there was a statistically significantly higher proportion of children who reported a smoker in their household (*P* = 0.008) and a higher proportion of households utilizing an indoor burning stove (*P* = 0.011) among those diagnosed with RSV-B compared with RSV-A (Table 1).

**Table 1:** Sociodemographic and clinical characteristics by respiratory syncytial virus subtype

Variables	All, *N* (%)	RSV-A, *n* (%)	RSV-B, *n* (%)	*P*-Value
Demographics	*N* = 147	*n* = 106	*n* = 41	
Female sex	79 (53.7)	56 (52.8)	23 (56.1)	0.864
Age in months, median [IQR]	5.8 [2.7 to 10.4]	6.5 [2.9 to 10.4]	5.0 [2.3 to 10.2]	0.503
Premature, <37 weeks	121 (82.3)	88 (83.0)	33 (80.5)	0.905
Breastfeeding in months, median [IQR]	5.0 [2.0 to 9.0]	6.0 [2.0 to 9.0]	5.0 [2.0 to 9.0]	0.740
Smoker lives in the household	40 (27.2)	23 (21.7)	17 (41.5)	0.008
Household uses an indoor stove	11 (7.5)	4 (3.8)	7 (17.1)	0.011
Clinical characteristics at enrollment				
BMI for age z-score [IQR]	−1.55 [–2.51 to –0.07]	−1.64 [–2.85 to –0.68]	−0.75 [–1.97 to 1.09]	0.002
Acute malnutrition				
Normal	98 (68.1)	64 (62.1)	34 (82.9)	0.037
Moderate	24 (16.7)	19 (18.4)	5 (12.2)	
Severe	22 (15.3)	20 (19.4)	2 (4.8)	
Poor feeding	68 (46.3)	42 (39.6)	26 (63.4)	0.016
Pneumonia	84 (57.1)	52 (49.1)	32 (78.0)	0.003
Bronchiolitis	99 (67.3)	71 (67.0)	28 (68.3)	1.000
Bronchitis	15 (10.2)	14 (13.2)	1 (2.4)	0.068
Laryngotracheobronchitis	4 (2.7)	1 (0.9)	3 (7.2)	0.066
Cough	146 (99.3)	105 (99.1)	41 (100.0)	1.000
Fast breathing for age	137 (93.2)	97 (91.5)	40 (97.6)	0.284
Nasal congestion	86 (58.5)	53 (50.0)	33 (80.5)	0.001
Nasal flaring	45 (30.6%)	36 (34.0%)	9 (22.0%)	0.223
Nasal flaring severity*				
Mild (flaring only)	24 (53.3)	23 (63.9)	1 (11.1)	0.009
Moderate (retraction also)	17 (37.8)	10 (27.8)	7 (77.8)	
Severe (accessory muscle use)	4 (8.9)	3 (8.3)	1 (11.1)	
Oxygen saturation %, median [IQR]	97.0 [96.0 to 98.0]	97.0 [96.0 to 98.0]	97.0 [96.0 to 98.0]	0.860
More than one virus detected^†^	45 (36)	33 (31.1)	12 (29.3)	0.984
Malaria RDT positive	14 (13.1)	10 (12.7)	4 (14.3)	0.757
HIV infection	1 (0.68)	1 (0.94)	0 (0.0)	1.000
Tuberculosis	1 (0.68)	0 (0.0)	1 (2.4)	0.279
Disposition				
Alive	132 (90.4)	97 (92.4)	35 (85.4)	0.334
Dead	2 (1.4)	1 (0.9)	1 (2.4)	
Referred	1 (0.7)	1 (0.9)	0 (0.00)	
Discharged against medical advice	8 (5.5)	5 (4.8)	3 (7.3)	

BMI = body mass index; RDT = rapid diagnostic test; RSV = respiratory syncytial virus; SARS-CoV-2 = severe acute respiratory syndrome coronavirus 2.

* Of children with nasal flaring (*n* = 45).

^†^ The viruses tested included adenovirus, human bocavirus, endemic human coronaviruses, human metapneumovirus, influenza A, B, and C, parainfluenza, human rhinovirus and enterovirus, respiratory syncytial virus, and SARS-CoV-2. Of children with a viral coinfection detected, none were diagnosed with SARS-CoV-2.

Clinically, malnutrition was common in the study population, with >30% of RSV-positive children classified as having either moderate or severe acute malnutrition. Comparing malnutrition across RSV subtypes, body mass index (BMI) z-scores were worse for children with RSV-A (–1.64 [IQR: –2.85 to –0.68]) compared with those with RSV-B (–0.75 [IQR: –1.97 to 1.09]; *P* = 0.002).

To rule out seasonality as the cause of the differences observed between RSV subtypes for BMI and household smoke exposure, both variables were compared against each of the RSV-A peak periods (cases before December 2021 versus cases on or after January 2022), and no significant differences were found (data not shown).

When the proportions of reported respiratory symptoms were compared across RSV subtypes, a statistically significantly higher proportion of children diagnosed with RSV-B were documented as having been diagnosed with pneumonia during their hospital stay (78% versus 49.1%; *P* = 0.003). Furthermore, although the incidence of bronchiolitis was similar across subtypes, both bronchitis and laryngotracheobronchitis exhibited a nonsignificant trend toward a higher proportion being diagnosed in the RSV-A group (*P* = 0.068 and *P* = 0.066, respectively). Nasal congestion was significantly more frequent in the RSV-B group (80.5% versus 50.0%; *P* = 0.001), as was moderate to severe nasal flaring (36.1% versus 88.9%; *P* = 0.009). Oxygen saturation levels at enrollment were within an appropriate range, with a median of 97% across subtypes (*P* = 0.860). There were no significant differences between the RSV subtypes in terms of the proportion of coinfection with other respiratory viruses, malaria, HIV, or tuberculosis (Table 1).

Of the patients admitted to the hospital with RSV, fewer than 2% died during their hospitalization, ∼90% were discharged alive, and the remaining 8% chose to leave the hospital before being discharged by the physician.

### Respiratory syncytial virus seasonality.

**Figure 2.**
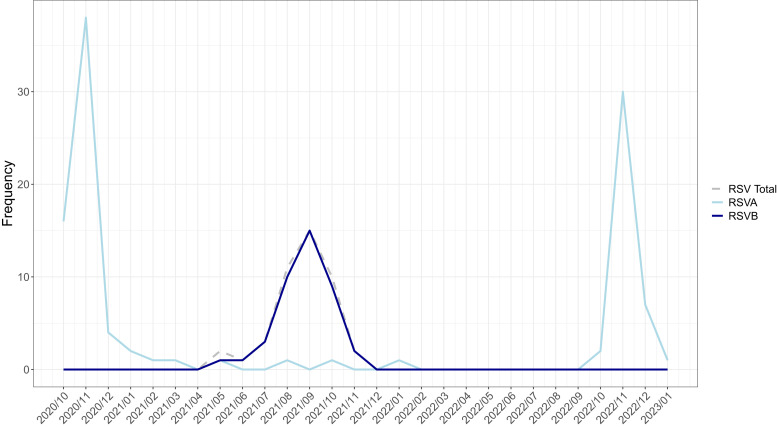
Seasonal distribution of respiratory syncytial virus (RSV)-A and RSV-B.

The present study is based on data collected over a 28-month period, slightly more than two consecutive respiratory seasons. During this period, RSV exhibited distinct peaks in cases corresponding to the months of October to December in all three years: 2020, 2021, and 2022. This timing corresponds with the wet season in Sierra Leone, which typically runs from May to November (Figure 2).^12^ Across the study period, RSV-A and RSV-B circulated during distinct and alternating years; RSV-A was the only subtype identified from October to December in 2020 and 2022, and RSV-B was predominant in 2021. In 2021, RSV-B exhibited fewer absolute numbers of cases compared with the years when RSV-A circulated; however, the total number of months of transmission was longer, spanning from May to November 2021.

### Need for oxygen therapy, referral to the ICU, and increasing severity score.

In multivariable analysis, patient characteristics associated with the need for O^2^ therapy, referral to a higher level of care in the ICU, and worsening respiratory disease severity scores were investigated (Table 2). Respiratory syncytial virus-B did not significantly impact the likelihood of requiring oxygen therapy, referral to the ICU, or disease severity. The characteristics most associated with a need for supplemental O^2^ therapy or referral to a higher level of care in the ICU were younger age at admission and having been born prematurely. For instance, the odds of requiring O^2^ therapy or being referred to the ICU reduced by 18% and 12%, respectively, for every 1-month increase in the child’s age (for O^2^ therapy: adjusted odds ratio [aOR]: 0.82; 95% CI: 0.74–0.89; *P* <0.001; for ICU referral: aOR: 0.88; 95% CI: 0.81–0.94). Children born prematurely had three- and twofold higher odds of requiring O^2^ therapy and being referred to the ICU, respectively (aOR: 3.1; 95% CI: 1.2–9.3; *P* = 0.041 for O^2^ therapy and aOR: 2.4; 95% CI: 0.9–6.6; *P* = 0.100 for ICU referral), although the latter did not reach statistical significance at the 5% level. Additionally, having more than one virus detected at admission was associated with a roughly twofold increased likelihood of needing O^2^ therapy (aOR: 2.3; 95% CI: 1.0–5.8; *P* = 0.065), although this did not reach statistical significance.

**Table 2:** Determinants of the need for oxygen therapy, referral to the intensive care unit, and worsening severity score

Variables	O^2^ Required		Referral to ICU		Severity Score	
	aOR (95% CI)	*P*-Value	aOR (95% CI)	*P*-Value	aOR (95% CI)	*P*-Value
RSV-B	1.21 (0.47–3.17)	0.697	0.90 (0.35–2.34)	0.827	0.51 (0.25–1.04)	0.063
Viral coinfection	2.30 (0.97–5.80)	0.065	1.01 (0.44–2.36)	0.978	1.42 (0.74–2.70)	0.291
Sex (male)	1.15 (0.51–2.62)	0.728	1.07 (0.47–2.42)	0.878	0.51 (0.27–0.95)	0.035
Age (per 1 month increase)	0.82 (0.74–0.89)	<0.001	0.88 (0.81–0.94)	0.001	0.82 (0.77–0.88)	<0.001
Indoor pollutants	0.69 (0.28–1.71)	0.429	0.67 (0.28–1.60)	0.364	1.86 (0.94–3.70)	0.076
Prematurity (<37 WGA)	3.06 (1.07–9.29)	0.041	2.35 (0.85–6.57)	0.100	1.20 (0.51–2.82)	0.684
GAM: moderate/severe*	0.92 (0.37–2.27)	0.851	0.40 (0.15–1.02)	0.138	1.13 (0.57–2.24)	0.725
GCM: moderate/severe^†^	0.73 (0.28–1.85)	0.503	1.08 (0.98–1.29)	0.059	1.08 (0.54–2.16)	0.835
Hospital stay (days)	1.06 (0.97–1.23)	0.341	0.86 (0.38–1.97)	0.290	1.00 (0.96–1.04)	0.971
Prior antibiotics^‡^	0.45 (0.19–1.03)	0.063	0.40 (0.15–1.02)	0.721	1.03 (0.56–1.90)	0.919

aOR = adjusted odds ratio; GAM = global acute malnutrition; GCM = global chronic malnutrition; ICU = intensive care unit; O^2^ = oxygen; RSV = respiratory syncytial virus; WGA = weeks gestational age.

* Weight for height z-score greater than –2.

^†^ Height for age z-score greater than –2.

^‡^ Prior antibiotics = antibiotics taken for this illness but before admission.

Similar to the need for O^2^ therapy and referral to the ICU, older children had lower odds of having an increased respiratory disease severity score (aOR: 0.82; 95% CI: 0.77–0.88; *P* <0.001). Comparably, children living in a home with either a smoker present or an indoor burning stove had a roughly twofold higher likelihood of having an increased disease severity score (aOR: 1.9; 95% CI: 0.9–3.7; *P* = 0.076); however, this did not reach statistical significance.

## DISCUSSION

In the study cohort of children under 24 months of age admitted to the hospital in Eastern Province, Sierra Leone, RSV accounted for ∼23% of all respiratory viral infections identified. The study findings align with those of studies conducted in Jordan and other Middle Eastern and North African regions in recent years, before the coronavirus disease 2019 (COVID-19) pandemic, which also revealed RSV to be a leading cause of respiratory illness in infants and young children.^1^^,^^2^^,^^12^ In contrast, the present study’s results may not align with those of global reports that highlighted a marked decline in RSV cases during the early pandemic years (2020–2021).^13^^,^^19^ Although prepandemic data for direct comparison are lacking, the RSV incidence observed in the study cohort suggests that its circulation may not have diminished as significantly in the study region.

One main hypothesis for the global reduction in RSV and other respiratory viruses is that COVID-19 mitigation strategies, such as wearing masks, practicing hand hygiene, and maintaining social distancing, have contributed to this decline. This hypothesis is further supported by reports of RSV resurgence in many regions as these measures relaxed in 2021.^20^^,^^21^ In contrast, the stability of RSV circulation in Kenema, Sierra Leone, may reflect regional differences in the implementation and adherence to these measures. In low-resource settings like Kenema, socioeconomic factors and crowded living conditions could make it challenging to implement and sustain public health measures effectively. Moreover, limited access to information and resources in rural areas may have hindered the widespread adoption of preventive practices, potentially contributing to the continued transmission of RSV, despite global trends.

Distinct seasonal patterns of RSV were observed in the present study, with sharp peaks in RSV cases occurring between October and December in 2020 and 2022, as well as between August and November in 2021. These peaks in RSV transmission aligned with Sierra Leone’s wet season—a finding consistent with other tropical regions—which is known to drive respiratory virus spread owing to increased indoor crowding and higher humidity levels.^13^^,^^19^ Epidemiologic studies have previously revealed that in tropical regions, the higher humidity and ambient temperatures during the wet season facilitate RSV survival in both aerosol and droplet forms. However, to date, no data specifically revealing the influence of humidity on RSV subtype-specific transmission exist.^20^^–^^22^

Examining RSV subtypes within the study cohort revealed that RSV-A was the most predominant across the entire study period, representing ∼70% of all RSV-positive samples. The predominance of RSV-A as the dominant circulating strain is well-documented in the literature, which often notes the cocirculation of RSV-A and RSV-B during any given season. The study findings revealed that nearly 100% of RSV cases that occurred from October to December 2020 and from October 2022 to January 2023 were caused by RSV-A; in contrast, RSV-B was more prevalent between July and October 2021.

This alternating dominance between RSV-A and RSV-B could be explained by viral interference, in which the active circulation of one subtype may suppress the transmission of the other. Variations in population immunity to the subtypes, potentially influenced by previous exposure, could also play a role in the changing dominance of these RSV subtypes. Such factors could result in shifts in subtype prevalence from year to year.^13^

In our cohort, younger age and prematurity were the most significant contributors to either one or all determinants of severe RSV outcomes. These findings are consistent with those of other RSV studies.^1^^,^^23^^,^^24^ Infants in their earliest months of life exhibited the greatest vulnerability to the virus, particularly those born prematurely. The median age of RSV-positive children was 5.8 months, and the data consistently revealed that with each additional month of life, the odds of requiring oxygen therapy or ICU care decreased. This finding underscores the developmental advantage that older infants have over younger ones as their immune systems and respiratory capacities mature. Prematurity further compounded the risk, with premature infants being three times more likely to need oxygen therapy, reinforcing their heightened susceptibility to severe disease.

Although malnutrition affected a significant proportion of our cohort, particularly those infected with RSV-A, it did not emerge as a significant driver of disease severity when adjusted for other variables. Although children with RSV-A exhibited poorer BMI z-scores compared with those with RSV-B (*P* = 0.002), this nutritional deficit did not correspond to worse clinical outcomes in terms of oxygen requirements or ICU referral. This finding suggests that although nutritional status remains an important overall health consideration, it does not independently increase RSV severity among hospitalized patients, particularly when other risk factors, such as age and prematurity, are more prominent. These findings are consistent with the results of Yanis et al. (2021),^13^ which revealed no significant differences in disease severity between RSV-A and RSV-B subtypes, despite RSV-A being more likely to present with decreased appetite and other clinical features. In the present study, nutrition status was only analyzed within the context of a cohort of children who required hospitalization. Data on nutritional status were not collected for children in the community, as it was outside the scope of the study. In this context, a limitation of the current study is the inability to determine whether a presumably poorer nutritional status was associated with a child’s increased likelihood of requiring hospitalization.

Comparing the two subtypes, RSV-B was more frequently associated with clinical presentations involving pneumonia and poor feeding. Children infected with RSV-B had a higher prevalence of pneumonia and poor feeding compared with those with RSV-A. These symptoms suggest a potentially more pronounced respiratory burden in RSV-B cases, despite the lack of significant differences in disease severity scores or outcomes, such as oxygen therapy or ICU referral. In contrast, children infected with RSV-A, although displaying poorer nutritional status, did not exhibit more severe disease outcomes compared with their RSV-B counterparts. This observation aligns with the findings of Toepfer et al. (2024), who also noted that although RSV-A was associated with higher hospitalization rates and supplemental oxygen use, these outcomes did not translate into more severe disease outcomes, such as ICU admission or the need for mechanical ventilation.^25^

Interestingly, although coinfections with other viruses were linked to increased oxygen requirements (aOR: 2.3; *P* = 0.065), they did not significantly influence the overall severity of the disease when compared with primary risk factors, such as age and prematurity. This highlights the complexity of managing respiratory illness in young children, particularly when multiple pathogens are present but still reinforces the predominance of biological vulnerability over viral subtype in predicting severe disease.

Contrary to the initial assumptions, indoor pollutants, such as household smoking or stove use, did not significantly contribute to increased disease severity. Although a higher proportion of RSV-B-positive children lived in households with smokers (41.5% versus 21.7%; *P* = 0.008), and more RSV-B-positive children came from homes using indoor stoves (17.1% versus 3.8%; *P* = 0.011), these environmental exposures did not correlate with more severe clinical outcomes. This suggests that although environmental pollutants may exacerbate respiratory symptoms in other contexts, they did not have a significant impact on RSV severity in this population, compared with factors such as age and prematurity.

The study findings reaffirm the importance of focusing clinical management on younger, premature infants, who consistently demonstrate a highest risk for severe RSV outcomes. Although there were notable differences in the clinical presentations of RSV-A and RSV-B, particularly in terms of pneumonia and nutritional status, these differences did not translate into differences in severity when adjusted for other factors. The biological vulnerability associated with age and prematurity remains the key driver of severe disease, indicating that the administrators of targeted interventions should prioritize these high-risk groups to improve outcomes.

The COVID-19 pandemic reshaped the epidemiology of respiratory infections; however, despite a global reduction in many studied respiratory viruses, RSV continued to circulate in the study area, with distinct seasonal peaks and alternating subtypes. This resilience, even amid pandemic restrictions, contrasts with the sharp declines in RSV reported in some regions, such as Australia and other parts of Africa, where measures temporarily suppressed RSV transmission.^12^ The study findings highlight the persistence of RSV and the need for sustained vigilance, even during broader public health crises.

The current study has several limitations. As an observational study, causal inferences are limited, and no adjustments were made for confounding factors, such as socioeconomic status or healthcare access, which might affect RSV transmission and severity. Additionally, by focusing only on hospitalized children, the findings may not reflect the full spectrum of RSV cases in the region, particularly outpatient care. The unique timing of the study during the COVID-19 pandemic also impacts the generalizability of the findings for future years, especially because RSV transmission may change post-pandemic. Furthermore, the 28-month observation period may not capture longer-term trends in RSV seasonality, and the limited bacterial culture capacity hindered the authors’ ability to investigate bacterial coinfections. Although the study was not designed to measure year-to-year differences in healthcare access, it is possible that health-seeking behaviors related to respiratory infections decreased during the COVID-19 pandemic. If so, this could have resulted in sicker children, such as those with pneumonia, being more likely to seek care, thus confounding the interpretation that RSV-B was associated with pneumonia during the 2021 season. However, pediatric clinicians at KGH anecdotally reported a low number of symptomatic COVID-19 cases overall throughout the 2020 and 2021 seasons. However, this did not result in a decrease in overall hospital admissions during this time period. Enrollment was also limited to the first three eligible patients per day; therefore, the true burden of RSV is underestimated in the current study. Additionally, demographic and other risk factors may influence comparisons.

## CONCLUSION

In conclusion, the present study underscores the critical risk factors for RSV, particularly young age, prematurity, and malnutrition. These findings reinforce the importance of targeted public health efforts, particularly for high-risk groups, as well as the need for ongoing development of effective vaccines and treatments to manage RSV in vulnerable populations.

## Supplemental Materials

10.4269/ajtmh.24-0845 Supplemental Materials

## References

[1] Shi T, ; RSV Global Epidemiology Network, 2017. Global, regional, and national disease burden estimates of acute lower respiratory infections due to respiratory syncytial virus in young children in 2015: A systematic review and modelling study. Lancet 390: 946–958. 28689664 10.1016/S0140-6736(17)30938-8 PMC5592248

[2] Nair H, , 2010. Global burden of acute lower respiratory infections due to respiratory syncytial virus in young children: A systematic review and meta-analysis. Lancet 375: 1545–1555. 20399493 10.1016/S0140-6736(10)60206-1 PMC2864404

[3] Colosia A Costello J McQuarrie K Kato K Bertzos K, 2023. Systematic literature review of the signs and symptoms of respiratory syncytial virus. Influenza Other Respir Viruses 17: e13100. 36824394 10.1111/irv.13100 PMC9899685

[4] Dawson-Caswell M Muncie HL Jr. 2011. Respiratory syncytial virus infection in children. Am Fam Physician 83: 141–146. 21243988

[5] Perk Y Özdil M, 2018. Respiratory syncytial virüs infections in neonates and infants. Turk Arch Pediatr Pediatri Arş 53: 63–70. 10.5152/TurkPediatriArs.2018.6939 PMC6089794 30116126

[6] Munro APS Martinón-Torres F Drysdale SB Faust SN, 2023. The disease burden of respiratory syncytial virus in Infants. Curr Opin Infect Dis 36: 379–384. 37610444 10.1097/QCO.0000000000000952 PMC10487373

[7] Kusel MMH de Klerk NH Kebadze T Vohma V Holt PG Johnston SL Sly PD, 2007. Early-life respiratory viral infections, atopic sensitization, and risk of subsequent development of persistent asthma. J Allergy Clin Immunol 119: 1105–1110. 17353039 10.1016/j.jaci.2006.12.669 PMC7125611

[8] United Nations Human Development Reports, 2024. *Country Insights*. Available at: https://hdr.undp.org/data-center/country-insights. Accessed June 26, 2024.

[9] Quinn M, 2016. *Governance and Health in Post-Conflict Countries: The Ebola Outbreak in Liberia and Sierra Leone*. Available at: https://papers.ssrn.com/abstract=2893312. Accessed June 26, 2024.

[10] World Health Organization, 2024. *Global Influenza Programme: Influenza Surveillance Outputs*. Available at: https://www.who.int/teams/global-influenza-programme/surveillance-and-monitoring/influenza-surveillance-outputs. Accessed June 26, 2024.

[11] Kebede S, , 2013. Establishing a national influenza sentinel surveillance system in a limited resource setting, experience of Sierra Leone. Health Res Policy Syst 11: 22. 23800108 10.1186/1478-4505-11-22 PMC3694480

[12] Samuels RJ, , 2023. Respiratory virus surveillance in hospitalized children less than two-years of age in Kenema, Sierra Leone during the COVID-19 pandemic (October 2020–October 2021). PLoS One 18: e0292652. 37816008 10.1371/journal.pone.0292652 PMC10564235

[13] Yanis A, , 2021. The clinical characteristics, severity, and seasonality of RSV subtypes among hospitalized children in Jordan. Pediatr Infect Dis J 40: 808–813. 34260483 10.1097/INF.0000000000003193

[14] Shaffer JG, ; Viral Hemorrhagic Fever Consortium, 2014. Lassa fever in post-conflict Sierra Leone. PLoS Negl Trop Dis 8: e2748. 24651047 10.1371/journal.pntd.0002748 PMC3961205

[15] Samuels RJ, , 2020. Lassa fever among children in Eastern Province, Sierra Leone: A 7-year retrospective analysis (2012–2018). Am J Trop Med Hyg 104: 585–592. 33241780 10.4269/ajtmh.20-0773 PMC7866338

[16] Moon TD Sumah I Amorim G Alhasan F Howard LM Myers H Green AF Grant DS Schieffelin JS Samuels RJ, 2023. Antibiotic prescribing practices for acute respiratory illness in children less than 24 months of age in Kenema, Sierra Leone: Is it time to move beyond algorithm driven decision making? BMC Infect Dis 23: 626. 37749485 10.1186/s12879-023-08606-0 PMC10519098

[17] Tal A Bavilski C Yohai D Bearman JE Gorodischer R Moses SW, 1983. Dexamethasone and salbutamol in the treatment of acute wheezing in infants. Pediatrics 71: 13–18. 6129609

[18] R Core Team, 2024. *R: A Language and Environment for Statistical Computing*. Available at: https://www.R-project.org/. Accessed August 31, 2025.

[19] Yassine HM Sohail MU Younes N Nasrallah GK, 2020. Systematic review of the respiratory syncytial virus (RSV) prevalence, genotype distribution, and seasonality in children from the Middle East and North Africa (MENA) Region. Microorganisms 8: 713. 32403364 10.3390/microorganisms8050713 PMC7284433

[20] Paynter S, 2015. Humidity and respiratory virus transmission in tropical and temperate settings. Epidemiol Infect 143: 1110–1118. 25307020 10.1017/S0950268814002702 PMC9507187

[21] Bloom-Feshbach K Alonso WJ Charu V Tamerius J Simonsen L Miller MA Viboud C, 2013. Latitudinal variations in seasonal activity of influenza and respiratory syncytial virus (RSV): A global comparative review. PLoS One 8: e54445. 23457451 10.1371/journal.pone.0054445 PMC3573019

[22] Divarathna MVM Rafeek RAM Morel AJ Aththanayake C Noordeen F, 2023. Epidemiology and risk factors of respiratory syncytial virus associated acute respiratory tract infection in hospitalized children younger than 5 years from Sri Lanka. Front Microbiol 14: 1173842. 37434712 10.3389/fmicb.2023.1173842 PMC10330818

[23] de Almeida RS Leite J Atwell JE Elsobky M LaRotta J Mousa M Thakkar K Fletcher MA, 2024. Respiratory syncytial virus burden in children under 2 years old in understudied areas worldwide: Gap analysis of available evidence, 2012–2022. Front Pediatr 12: 1452267. 39639952 10.3389/fped.2024.1452267 PMC11617186

[24] Tempia S, , 2017. Risk factors for influenza-associated severe acute respiratory illness hospitalization in South Africa, 2012–2015. Open Forum Infect Dis 4: ofw262. 28480255 10.1093/ofid/ofw262 PMC5414019

[25] Toepfer AP, , 2024. Seasonality, clinical characteristics, and outcomes of respiratory syncytial virus disease by subtype among children aged <5 years: New vaccine surveillance network, United States, 2016–2020. Clin Infect Dis 78: 1352–1359. 38366649 10.1093/cid/ciae085 PMC11093674

