# Supplementary material for: Respiratory Syncytial Virus Epidemiology and Clinical Characteristics among Young Children Hospitalized in Sierra Leone
Source: Am J Trop Med Hyg. 2025 Sep 4;113(5):1057–63. doi: 10.4269/ajtmh.24-0845 (PMC12590980; doi:10.4269/ajtmh.24-0845)
Supplement: Supplemental Materials [file tpmd240845.SD1.pdf]

**Supplemental File:  Adapted Severity Score**

**O2 saturation**:

- 0, if O2 sat ≥95
- 1, if O2 sat is between [94,97]
- 2, if O2 sat is between [90,94]
- 3, if O2 sat <90

**Respiratory Rate**:

- 0, if respiratory rate <40
- 1, if respiratory rate is between [40,49]
- 2, if respiratory rate is between [49,59]
- 3, if respiratory rate >59

**Nasal flaring/use of accessory muscles**:

- 0, if no nasal flaring/use of accessory muscles present
- 3, if yes nasal flaring/use of accessory muscles present

**Wheezing**:

- 0, if no wheezing present
- 3, if yes wheezing present
